# Genome Sequence of *Clostridium botulinum* Strain Adk2012 Associated with a Foodborne Botulinum Case in Tottori, Japan, in 2012

**DOI:** 10.1128/genomeA.00872-17

**Published:** 2017-08-24

**Authors:** Hiroshi Asakura, Shiori Yamamoto, Yoshika Momose, Haru Kato, Masaaki Iwaki, Keigo Shibayama

**Affiliations:** a Division of Biomedical Food Research, National Institute of Health Sciences, Tokyo, Japan; b Department of Bacteriology II, National Institute of Infectious Diseases, Tokyo, Japan

## Abstract

We report here a draft genome sequence of *Clostridium botulinum* Adk2012 responsible for a foodborne botulism case that occurred in Tottori, Japan, in 2012. Its genome size was 2,904,173 bp, with 46 rRNAs and 54 tRNAs, at a coverage of 14.5×.

## GENOME ANNOUNCEMENT

*Clostridium botulinum* is a spore-forming anaerobe with the ability to produce botulinum neurotoxin (BoNT) that causes a neuroparalytic disease, botulism (1, 2). Although the incidence of botulism is not frequent, this pathogen is recognized as one of the most serious biological hazards because of its high mortality rate (3). The most common form in Western countries is infant botulism, while foods also occasionally transmit this pathogen to humans through the ingestion of BoNT generated in the food matrixes. Typically, home-canned food substances and fermented uncooked dishes are thought to be some of the major sources for foodborne botulism (http://www.cdc.gov/botulism).

On March 2012, one foodborne botulism case occurred by the intake of contaminated adzuki batto, a sweet adzuki bean soup containing flat wheat noodles in a vacuum package (4). Although the causative *C. botulinum* strain, Adk2012, harbored both *ha33* and *p47* genes, representing the BoNT/A and BoNT/B clusters, respectively (5), only BoNT/A was detected from the causative food at 75,000 50% lethal dose (LD_50_)/g of weight intraperitoneally (i.p.) in a mouse toxicity assay (4).

To further characterize its genomic trait(s), this study determined a draft genome sequence of the *C. botulinum* Adk2012 strain originating from the causative food of a foodborne botulism case in Japan 2012. The genomic DNA of the strain was extracted using the Genomic tip-500G kit (Qiagen, Hilden, Germany), followed by sequencing analysis using paired-end sequencing with a FLX Titanium sequencer (Roche, Basel, Switzerland), which resulted in an average coverage of 14.5×. Raw reads were trimmed and *de novo* assembled using CLC Genomics Workbench version 9.5 (Qiagen). The parameters for trimming were as follows: ambiguous limit, 2; quality limit, 0.05; number of 5′-terminal nucleotides, 20; and number of 3′-terminal nucleotides, 5. The parameters for *de novo* assembly were as follows: mapping mode, create simple contig sequences (fast); bubble size, 50; word size, 21; minimum contig length, 2,000 bp; perform scaffolding, no; and autodetect paired distances, yes. The draft genome of the *C. botulinum* Adk2012 strain was assembled into 275 contigs, with an accumulated length of 2,904,173 bp (*N*_50_, 12,881 bp) and an average G+C content of 28.4%. The genome was annotated by the RAST server (6). Annotation of these assemblies identified 3,486 coding sequences (CDSs), 46 rRNAs, and 54 tRNAs.

The nucleotide sequence of the BoNT/A gene in the Adk2012 strain showed 100% identity to the BoNT/A1 in strain ATCC 3502, while the sequence of BoNT/B1 gene contained stop codons and deletions, indicating its loss of function, which was in agreement with a previous finding by Hutson et al. (7). The SEED Viewer program also showed that the *C. botulinum* A strain ATCC 3502 was one of the closest neighbors to the Adk2012 strain. Considering the genomic diversity of this pathogen (8), further accumulation of genomic data will provide new information on pathogen biology and transmission and inform studies on pathogen evolution.

### Accession number(s).

This whole-genome shotgun project has been deposited at DDBJ/EMBL/GenBank under the accession number BDQO00000000. The version described in this paper is the second version, BDQO02000000.
